# Ankylosis

**Published:** 1900-04

**Authors:** N. F. Howard


### CORRESPONDENCE.

---

### ANKYLOSIS.

DAHLONEGA, GA., February, 1900.

*To the Editor of the Atlanta Journal-Record of Medicine:*

When Dr. F. W. McRae, of Atlanta, was in Dahlonega in 1899, to see a little patient with hip-joint disease, he made the statement in substance that if a healthy joint was confined and bound and kept in this state in an easy position for months and even years, ankylosis would not take place in said joint, but if unbound and set free it would assume and perform its healthy and normal functions, and that ankylosis could only take place when inflammation or a diseased condition of the joint existed. This remark of Dr. McRae's brought up to my mind many cases of persons who claimed to have stiff joints but did not. Permit me to state one case that will illustrate many others, to wit: When at Vicksburg in 1863, as surgeon of the fifty-second Georgia regiment, a soldier, Private ——, who had been absent many months without leave, was returned to his command and made application for a discharge from the army on the ground of disability to perform military duty. The medical officers investigated his case and examined his condition. We found one arm carried in a swing bandage. The soldier complained of rheumatism and pain and stiffness of said arm from his shoulder to his hand. The skin was tender and of a flesh color, and the arm had the appearance of having been in a bandage for many months, and the hand and forearm were brought across the chest at a right angle with his arm. He claimed the elbow was stiff, and that it gave him great pain to try to straighten the arm. We made the effort to do so, however, and we found that it took more doctors than one to do anything with him. He made such complaint of the pain and suffering that it gave him, that he was turned loose and put on light duty, which was to look after the camp and so forth. As soon as this soldier could do so, he left the command and went to the other side. Two years after this, when peace was concluded, we met him in Dahlonega and found that his arm had been restored to a state of perfect health, as we had observed in many other cases thus affected.

### A DWARFED MEMBER OR ORGAN OF THE HUMAN BODY.

I ask this question : Will a member or organ of the human body, bound and kept in that position from childhood to a state of maturity, if unloosed and set at liberty, grow and increase in bulk so as to assume the normal functions of full-grown manhood? We know that in the case of the solid organs of the body such as bones, they will not, as is shown in the treatment of the feet and heads of the children of Chinamen; but we will ask about the lobes and soft organs of the body, as the liver, lungs, tongue and others. Here we wish to state the case of a tongue, dwarfed in childhood. This was a grandchild of ours twenty months old. His mother brought him to our house at that time to remain with us, and claimed that the child was tongue-tied, and wished us to clip the frenum of the tongue, so that the child might talk. On examination of the tongue, we found the mouth was full of well-set teeth, but that the tongue was very small and short and could not be protruded beyond the teeth, and that the child could utter but very few words, such as pa, ma, eat and other very simple words. On inquiry we learned that the child had been fed by hand, as it could not nurse its mother. At the age of sixteen months this boy, Howard Stonewall Howard, was taught by his parents to drink milk from the cup and give up the bottle; but as soon as he would eat he would cry for his rubber nipple, and he kept it in his mouth and nursed it from one meal to another. I decided that this was the cause of the tongue's being so dwarfed, and directed that he should not be permitted to use the rubber nipple any more. This direction was complied with. In one month a very perceptible improvement was discovered in his speech, and he could utter many words. The improvement has continued up to this time, and now he can talk as well as any child you may meet with, two years old, and we find, on examination of the tongue, that it is well grown in size and length, and has no need of being clipped for being tongue-tied, as it can now be protruded well out of the mouth beyond the lips.

Do not we physicians often commit an error in clipping the tongue at too early an age, and not giving it a fair chance to grow and overcome the seeming trouble? My fifty years' experience and observation teach me that fact.

Now we ask again, would these soft organs, such as the tongue, after being kept bound till maturity, regain their wonted size and usefulness?

We have seen children suck their thumbs until they were well-nigh grown, and the thumb they sucked would be one-third smaller than the other; but we are not informed whether the dwarfed thumb ever grew enough to catch up in size and usefulness with the other.

We have written this communication to call attention to the subject of ankylosis and a dwarfed condition of the soft parts of the human body, and to ask the brethren of our profession to give attention to this subject in their practice with children.

I trust you will give it a place in THE JOURNAL.

N. F. HOWARD, M.D.

